# Enhancement of Electricity Production in Microbial Fuel Cells Using a Biosurfactant-Producing Co-Culture

**DOI:** 10.3390/molecules28237833

**Published:** 2023-11-29

**Authors:** Carolina Montoya-Vallejo, Jorge Omar Gil Posada, Juan Carlos Quintero-Díaz

**Affiliations:** Grupo de Bioprocesos, Departamento de Ingeniería Química, Universidad de Antioquia, Medellín 050010, Colombia; carolina.montoya1@udea.edu.co (C.M.-V.); jomar.gil@udea.edu.co (J.O.G.P.)

**Keywords:** *Lactobacillus*, co-culture, Tween 80, surfactin, *E. coli*

## Abstract

Microbial fuel cells are bio-electrochemical devices that enable the conversion of chemical energy into bioelectricity. In this manuscript, the use of biosurfactants (Tween 80 and surfactin) and the effect of coculturing *E. coli* and *L. plantarum* were used to investigate the generation of bioelectricity coming from an H-type microbial fuel cell. In this setup, *E. coli* acts as an electron donor while *L. plantarum* acts as an in situ biosurfactant producer. It was observed that the use of exogenous surfactants enhanced electricity production compared to conventional *E. coli* cultures. The utilization of Tween 80 and surfactin increased the power generation from 204 µW m^−2^ to 506 µW m^−2^ and 577 µW m^−2^, respectively. Furthermore, co-culturing *E. coli* and *L. plantarum* also resulted in a higher power output compared to pure cultures (132.8% more when compared to using *E. coli* alone and 68.1% more when compared to using *L. plantarum* alone). Due to the presence of surfactants, the internal resistance of the cell was reduced. The experimental evidence collected here clearly indicates that the production of endogenous surfactants, as well as the addition of exogenous surfactants, will enhance MFC electricity production.

## 1. Introduction

**Figure 1:**
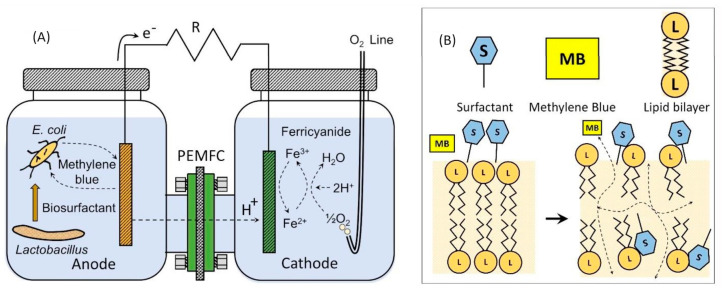
Microbial fuel cell for electricity production and surfactant mechanism on wall cell of bacteria. (**A**) *L. plantarum* and *E. coli* co-culture interaction within an MFC. (**B**) The interaction of surfactant with cell membrane will facilitate redox mediator (MB) permeation.

Microbial fuel cells (MFCs) are bio-electrochemical systems that convert chemical energy derived from organic matter into bioelectricity by redirecting the electron transport chain of a microorganism towards an external electric circuit [1], as can be seen in Figure 1A.

Modifications of electrode materials and structure, the customization of bacterial culture, and the optimization of MFC geometry and design have been demonstrated in recent advances to notably boost MFC power density. Nevertheless, the substantial limitation in MFC power density remains a primary obstacle that hampers the extensive implementation of this technology on a larger scale [2,3].

To improve MFC power generation, it is imperative to increase the speed of electron transfer from the microorganisms to the anode. Electron transfer can be achieved by using redox mediators, especially when non-electroactive microorganisms such as *E. coli* are used [4,5]. Redox mediator species, such as methylene blue (MB), methyl viologen (MV), anthraquinone-2,6-disulfonate (AQDS), 2-hydroxy-1,4-naphthoquinone, and resazurin, must be capable of penetrating the cellular membrane to receive electron charges from the cell. Subsequently, they can exit the cell and transfer electrons to the anode [6,7].

An increase in electron transfer can also be achieved by using surfactants, and these surface-active molecules can modify the microstructure of the cell membrane by forming channels [8]; this will, in turn, enhance the permeability of microbial cells, thus reducing their resistance to the transport of substrates and mediators (Figure 1B). It has been reported that the addition of surfactants to the anodic chamber of an MFC has rendered significant improvements in its overall power density. For example, a 2-fold power density increment was found when using tween 80 [9]; similarly, a staggering 8-fold increment in power density was reported when using EDTA [10]. Other surfactants that are more biocompatible with microorganisms, such as rhamnolipids, sophorolipids, and trehalose lipids, have also been used for power generation [11,12]. However, surfactants can also act as carbon sources for microbial growth, so their action will decrease over time [13,14]. Moreover, some synthetic surfactants may exhibit antibacterial effects [15], and their application in the environment would entail additional operational costs. For this reason, the in situ production of surfactants could be a solution. This strategy has been implemented through the overexpression of the *rhlA* gene responsible for rhamnolipids synthesis in *P. aeruginosa*, where the overall power output corresponded to almost 2.5 times the power density of the parent strain [16]. Synergistic collaboration between two microbial species has also been studied to increase the electricity production in Microbial Fuel Cells. *E. coli* and *Geobacter sulfurreducens* enhanced MFC performance due to *E. coli* biorreactor oxygen depletion [17]. Co-cultures between *E. coli* and *Pseudomonas aeruginosa* have improved power generation up to 37% when compared to the pure *E. coli* culture, thanks to the production of several redox mediators by *Pseudomona* [18,19]. Similarly, with cocultures of *P. aeruginosa* and *Klebsiella variicola*, the metabolite 1,3 propanediol produced by *Klebsiella variicola* induces the production of the mediator pyocyanin of *P. aeruginosa*, which increases the production of current up to three times when compared against a single culture [20]. To the best of the authors knowledge, there have been no studies where surfactant-producing microorganisms were cocultured with electricity-generating microorganisms within the anodic chamber of an MFC.

In this manuscript, the effect of adding exogenous surfactants, one synthetic (tween 80) and one biological (surfactin), to MFC electricity production by using *E. coli* as the anodic culture and methylene blue as a redox mediator was evaluated. Additionally, co-culturing *E. coli* (an electron donor) and *L. plantarum* (an endogenous source of biosurfactants) was used to enhance the MFC current generation. The novelty of this manuscript lies in enhancing the electricity production in microbial fuel cells by employing both exogenous and endogenous surfactants through a co-culture approach. In this approach, *Lactobacillus* produces a biosurfactant in situ, while *E. coli* degrades organic matter to generate electrons that are transferred to the anode via methylene blue as a redox mediator. The biosurfactant permeabilizes the cell wall of *E. coli* and promotes an increased electron flux towards the anode, thereby improving the performance of the MFC. To the best of the authors’ knowledge, this type of synergy, as found in the indicated co-culture, has not been previously studied in the literature.

## 2. Results and Discussion

**Figure 2:**
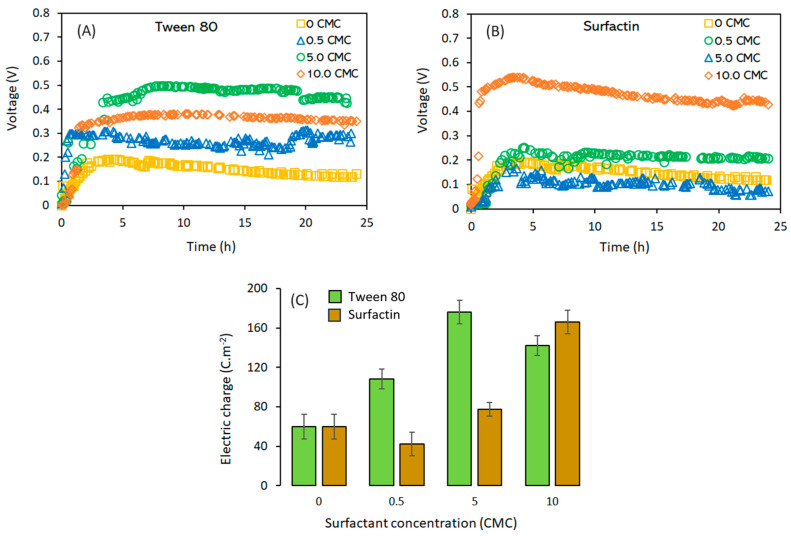
Voltage profiles and electric charge density for MFC with *E. coli* using 0.3 mM of methylene blue and 5.0 g L^−1^ of glucose. (**A**) Tween 80 voltage profile. (**B**) Surfactin voltage profile. (**C**) Electric charge density comparison.

Time domain voltage profiles for MFCs with the addition of selected surfactants, either surfactin or tween 80, are shown in Figure 2. The largest voltage values developed when using tween 80 at 0.5 CMC, 5.0 CMC, and 10 CMC (where CMC denotes the critical micelle concentration) were 0.31 V (1.4 mA m^−2^), 0.50 V (2.3 mA m^−2^), and 0.40 V (1.8 mA m^−2^), respectively, while, for surfactin, the maximal voltage values were 0.18 V (0.8 mA m^−2^), 0.25 V (1.1 mA m^−2^), and 0.54 V (2.4 mA m^−2^) for 0.5 CMC, 5.0 CMC, and 10 CMC, respectively. As shown on Figure 2, after approximately 5.0 h of treatment, the current reached a value that remained almost constant until the end of the fermentation. Figure 2C also provides a graphical comparison among the electric charge densities achieved by using either of the two surfactants. For Tween 80, a significant increase (*p* < 0.05) in electrical charge was observed for all the evaluated concentrations compared to the *E. coli* culture without surfactant. However, at 10 CMC, a reduction in cell behavior was observed, possibly due to an inhibition phenomenon generated by the synthetic surfactant. The use of tween 80 increased the electric charge density, reaching its larger value of 176.2 C m^−2^ at 5 CMC of tween 80; however, any further increase in the concentration of the surfactant will decrease the electric charge density. For example, when the concentration of tween 80 was held at 10 CMC, the charge density decreased to 166.1 C m^−2^. Tween 80 is an ionic surfactant that has been used to improve the performance of microbial fuel cells. Inhibition of *E. coli* DH5α growth when using tween 80 was found during the experiments; in addition, when the concentration of tween 80 was increased from 5 CMC to 10 CMC, the cell count decreased from 2.65 × 10^8^ CFU mL^−1^ to 1.73 × 10^8^ CFU mL^−1^, respectively.

On the other hand, no significant differences in electric charge were found at low concentrations of surfactin compared to the *E. coli* treatment without surfactant, indicating that, at this low concentration, no effect was observed on the cell’s behavior; for example, at 0.5 CMC of surfactin, the electric charge density was almost the same as the one measured for the MFC using only *E. coli* without any addition of surfactant. Unlike with tween 80, no inhibition was found when using large concentrations of surfactin. The electrical charge density increased as the surfactin concentration was increased, reaching its highest value at a concentration of 10 CMC. The largest charge density in the MFC was achieved by using tween 80 at 5 CMC and surfactin at 10 CMC, with values of 176.2 and 166.1 cm^−2^, respectively. No significant difference was observed between these two values (*p* > 0.05).

It has been claimed that the use of surfactants will improve the formation of transmembrane channels while enhancing the permeability of the cellular membrane (Figure 1B), thus promoting the transport of substrates and facilitating their degradation [12]. Similar observations have been reported when using selected surfactants with microbial fuel cells. For example, sodium dodecyl sulfate was used at a concentration of 5.0 mM with an MFC for the treatment of an anaerobic sludge, and it was found that, due to the surfactant’s biocompatibility, the hydrophilic properties of the anode were improved, the sludge adaptation period was shortened, and a high steady-state voltage was developed while rendering a maximum power output of 1640 mW m^−2^, which outperformed the control treatment (without surfactant) by almost 20%; however, increasing the concentration of sodium dodecyl sulfate above 10.0 mM had some major drawbacks, such as extended starting periods for the MFC and an increased electrode activation resistance, thus limiting the maximum power output of the MFC [21]. It has been claimed that tween 80 can be used with bacterial cultures to foment the passage of compounds through cellular membranes [9,11]. However, the microbial strain, as well as the surfactant concentration, might impact the transference of electrons. For example, when using *Geobacter sulfurreducens* with the surfactant tween 80, no enhancements in the production of current were noted [22]; however, different results were obtained when using microbial consortiums with a single-chamber MFC, where the power density was increased by almost 8.7 times with the mere addition of 80.0 mg L^−1^ of tween 80. In this study, a concentration of 10 CMC of tween 80 allowed for an observation of growth limitation and a decrease in current production. Power density was modelled by using a Monod-type kinetic model as a function of the concentration of tween 80, thus obtaining a half saturation constant Ks = 18.0 mg L^−1^, which indicates that this surfactant limits cell performance only at low concentrations [9]. Similarly, the surfactant Sophorolipids was used to enhance the performance of an MFC utilizing *Pseudomonas aeruginosa*, where current and power densities were increased 70% and 60%, respectively; likewise, an improvement in the MFC overall performance was noted and attributed to the in situ production and excretion of the mediator pyocyanin and to the increased membrane permeability [11]. On the other hand, it has been shown that the use of sophorolipid will reduce the internal resistances of an MFC up to 40% [16]. Reductions in the same parameter in the order of 30% have been reported when using *P. aeruginosa*, which endogenously overexpressed rhamnolipid [16].

These observations align with the experimental evidence gathered here, revealing reductions in internal resistance of approximately 65% and 70% when using Tween 80 and surfactin, respectively, compared to the control treatment (*E. coli* culture without any surfactant addition).The improvement in MFC electric charge density production due to surfactant addition was also explained by an increase in the bacteria’s electroactive surface area, which improves the transference of electrons to the anode. This transference is related to the bacteria–anode interaction, which is influenced not only by electrostatic and Van der Waals type interactions, but also by the hydrophobicity of the bacteria [21].

Biosurfactants are produced by a plethora of microorganisms, including *Pseudomona aeruginosa*, *Candida bombicola*, *Bacillus subtilis*, and a large number of *Lactobacillus*. Once produced, biosurfactants can be excreted to the medium [23]. By considering that biosurfactants favor cellular membrane permeability, this manuscript was conceived to evaluate the presence of *Lactobacillus plantarum*, a lactic acid bacteria, which has been previously evaluated for its ability to produce biosurfactants which do not inhibit *E. coli* growth [24].

**Figure 3:**
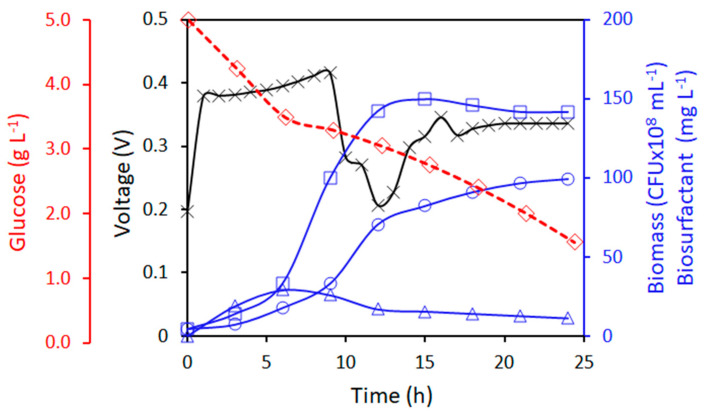
Kinetic profile of co-culture between *E. coli* DH5 α and *L. plantarum* in anodic chamber of MFC at 5.0 g L^−1^ of glucose and 0.3 mM of methylene blue. Symbols: Voltage (black line ×), *L. plantarum* biomass (blue line **□**), *E. coli* biomass (blue line Δ), surfactant (blue line ○), and glucose (red line ◊).

The results of voltage evolution and microbial growth for a co-culture of *Lactobacillus plantarum* and *E. coli* DH5α are shown in Figure 3. During the first hour of operation, there is a monotonic growth, where the MFC renders 400 mV; this voltage remained relatively constant for almost 7 h, after which, the voltage dropped to almost 200 mV, a moment in which not only was there a reduction in the growth of *E. coli*, but also an exponential increment in the growth of *L. plantarum* took place. After 12 h of operation, the voltage increased to 330 mV, and this voltage value remained almost constant until the end of the treatment (24 h). After a 24 h run, the production of biosurfactants reached almost 100 mg L^−1^, which is somewhat smaller than what has been reported by other researchers that used richer microbial broths [24]. *L. plantarum* reached its maximum biomass concentration after almost 15 h of treatment, where a concentration of 140 × 10^8^ CFU mL^−1^ was reached, and this concentration remained almost constant until the end of the co-culture treatment, while *E. coli* reached its maximum biomass concentration after 7 h of co-culture at 29 × 10^8^ CFU mL^−1^; after this, its concentration decreased, reaching 11 × 10^8^ CFU mL^−1^.

This phenomenon of the potential inhibition of *E. coli* growth due to the presence of *L. plantarum* can be explained by the presence of lactic acid produced by *lactobacillus* [25]. Some weak acidic species, such as lactic acid, can diffuse through the cell membrane to finally dissociate, and this would render relatively toxic environments to *E. coli* [26,27]. It has been observed that co-cultures of *Lactobacillus casei* and *E. coli*, with similar bacterial counts at the earlier stages of the test, evolved in such a way that, after 24 h of treatment, cell counting was dominated by *L. casei* [28]. This was explained in terms of the adjacent-possible ecological niche. This concept hinges on the fact that microbial species can modify the environments they live in, and this would make those environments more suitable for species that otherwise would barely cope with them [28]. Finally, models where *Lactobacillus* species and *E. coli* compete for the same nutrients have been proposed; however, the usefulness of these models is very limited in predicting the *E. coli* bacterial growth [25].

**Figure 4:**
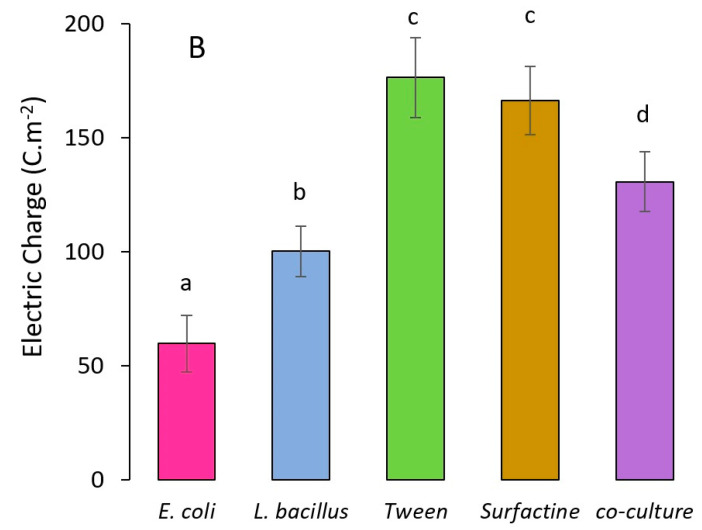
Electric charge generation at different surfactant conditions: all assays were conducted at 5.0 g L^−1^ of glucose, 0.3 mM of methylene blue, tween 80 at 5.0 CMC, and surfactin at 10.0 CMC. Lowercase letters a, b, c, d indicate significant differences between the treatments (*p* < 0.05).

Aiming to evaluate the electric charge density generation, the following conditions were compared, as illustrated in Figure 4: (i) culture of *E. coli* at 0.3 mM methylene blue (control). (ii) Culture of *L. plantarum* at 0.3 mM methylene blue. (iii) Culture of *E. coli* at 0.3 mM methylene blue and 5.0 CMC of Tween 80. (iv) Culture of *E. coli* at 0.3 mM methylene blue and 10.0 CMC of surfactin. (v) Co-culture of *E. coli* and *L. plantarum* at 0.3 mM methylene blue. Lowercase letters show that the means of each result were significantly different from each other (*p* < 0.05), except between Tween 80 at 5 CMC and surfactin at 10 CMC.

The first observation that can be drawn from these results relates to *L. plantarum*’s ability, in addition to producing biosurfactants, to transfer electrons to the anode and generate electricity. The electrical charge density produced was 67.8% higher than that obtained with the *E. coli* control culture. It was demonstrated that, in glucose-containing cultures, *L. plantarum* exhibits electrogenic activity mediated by type II NADH-quinone oxidoreductase [29]. Other *Lactobacillus* species such as *L. pentosus* and *L. casei* have also been employed for electricity production in microbial fuel cells [30].

As shown in Figure 4, the co-culture of *E. coli* and *L. plantarum* improved the electric charge density over the *E. coli* control treatment up to 118.9% (130.6 C m^−2^). This result demonstrates that, despite the dominance of *Lactobacillus* in the environment, a synergy was observed between the two species regarding electricity production, which was higher compared to the treatments with individual microorganisms. A similar strategy for operating microbial fuel cells using a co-culture of electricity-producing strains and biosurfactant-producing strains has not been reported in the literature.

The co-culture treatment was, however, outperformed by the use of exogenous surfactants, which, in the case of tween 80, was 195.5% higher (176.2 C m^−2^) and the use of surfactin was 178.5% higher (166.1 C m^−2^). The lower current generation obtained by co-culturing compared to using exogenous surfactants could be explained in terms of reduced levels of endogenously produced surfactants (in the order of 0.33 CMC) [24] compared to the concentration levels of the exogen surfactants that were added to the nutrient-rich microbial broth (5.0 CMC and 10 CMC for tween 80 and surfactin, respectively). It has been found that selected strains of *Lactobacillus* produce endogenous biosurfactants; the use of such strains in the anodic chamber rendered large open-circuit potential values in the order of 439 mV. For comparison, the use of *Lactobacillus* strains incapable of producing biosurfactants rendered open-circuit potential values in the order of 276 mV [16].

All of these findings evidence the synergy between *E. coli* and *L. plantarum*, as biosurfactant production and electron transport are produced to a larger extent when both strains work together. Co-culture synergies are not uncommon with MFC, for example, the use of *E. coli* and *Geobacter sulfurreducens* enhanced MFC performance due to *E. coli* biorreactor oxygen depletion [17]. It has been noted that *Pseudomona aeroginosa* is capable of producing several redox mediators, which makes this strain a good candidate for coculturing [18]. For example, co-cultures of *E. coli* and *Pseudomonas aeroginosa* improved power generation up to 37% when compared to the pure *E. coli* culture [19]. Similarly, with cocultures of *P. aeruginosa* and *Klebsiella variicola*, the metabolite 1,3-propanediol that is produced by the *Klebsiella variicola* induces the production of the mediator pyocyanin of *P. aeruginosa*, which increases the production of current up to three times when compared to a single culture [20]. Due to the complexity of microbial communities, there are still challenges in understanding population dynamics and their specific roles during substrate oxidation and electron transfer in bioelectrochemical process.

**Figure 5:**
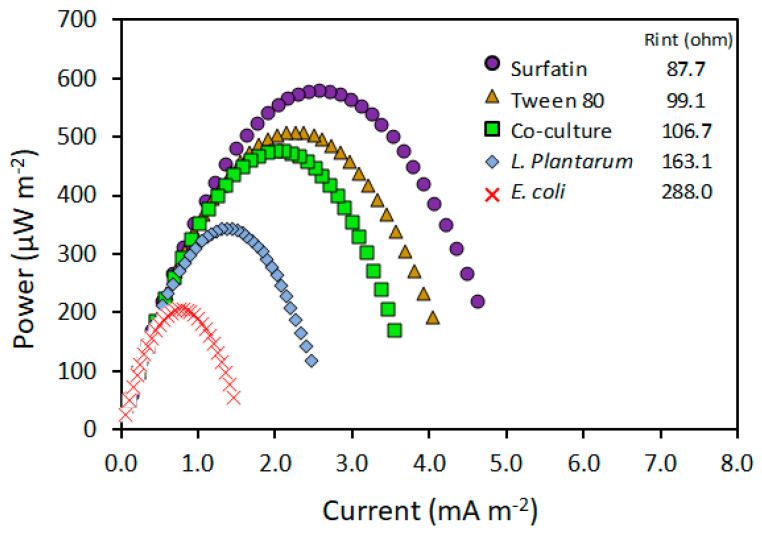
Power curves for MFC with *E. coli* as control using 0.3 mM of methylene blue and 5.0 g L^−1^ of glucose.

As indicated in Figure 5, during MFC operation with *E. coli*, the maximum power density achieved by the cell was 204.5 μW m^−2^ with an internal resistance in the order of 288.8 ohms. The addition of surfactin and tween 80 favored the generation of power of almost 282% (577 μW m^−2^) and 248% (506 μW m^−2^), respectively, when comparing against the control treatment with *E. coli*, this was also reflected by a drastic reduction in internal resistance (70% and 65%, respectively). This could be due to a more effective transport of electrons coming from the cell to the anode. On the other hand, the power density generated during the co-culture was in the order of 475 μW m^−2^, which is almost 2.3 times larger than the one obtained for the control treatment (with an internal resistance in the order of 106.7 ohms). Experiments with a biosurfactant-producing strain of *P. aeruginosa* compared to a non-producing strain also showed a significant reduction in internal resistance during the operation of a microbial fuel cell [16]. With microorganisms that do not experience significant competition during growth, it would be possible to use co-cultures to extend the operational period of a microbial cell without the need for the addition of exogenous surfactants and increase the obtained power. The presence of exogenous or endogenous surfactants represents an improvement in the electricity production in microbial fuel cells. The use of co-cultures is undoubtedly an alternative that allows for the creation of synergies between the biological capabilities of two microbial species to achieve the goal of enhancing the efficiency of such devices. Improving the efficiency of electricity production and its scalability will allow for wastewater treatment and the generation of useful electrical energy from its organic matter, suitable for domestic use, for example. However, fuel cells should not be limited to just these two applications. Other applications include the degradation of environmental contaminants, hydrogen production, biosensor design, biomolecule production, and CO_2_ capture, among others [31].

## 3. Materials and Methods

### 3.1. Reactor Design and Operation

*E. coli* DH5α was used as an anodic microorganism and was cultured in LB (Luria-Bertani) medium that contained 5.0 g L^−1^ of NaCl, 10 g L^−1^ of tryptone, and 5.0 g L^−1^ of yeast extract. The carbon source used was glucose at 5.0 g L^−1^.

A dual-chamber H-type microbial fuel cell was used to conduct experiments. The basic setup consisted of two glass chambers, each with a total capacity of 250 mL, separated by a Zirfon^®^ proton exchange membrane with dimensions of 1.5 cm × 1.5 cm. Graphite brush electrodes were used for both anodic and cathodic chambers (2.5 cm in outer diameter and 2.5 cm long, an average fiber diameter of 0.72 μm, and total area of 0.22 m^2^, MILL ROUSE). The anodic chamber was loaded with an *E. coli* DH5α suspension in LB medium that was supplemented with glucose as an electron donor and methylene blue at 0.3 mM as the redox mediator [32]. The cathodic chamber was filled with a solution of 20 mM K_3_[Fe(CN)_6_] and oxygen was supplied through air bubbling using an aquarium pump at a constant flow. An external resistance of 1 kΩ was part of the external circuit that connects the anode and cathode. Data acquisition and electrochemical measurements (polarization curves and voltage profiles) were performed by using a multi-channel potentiostat with FRA capabilities (MultiPalmSense4, Palmsens). A co-culture assay was also used to further investigate the effects of the mixed bacterial culture between *E. coli* and *L. plantarum* ATCC 8014, on the MFC of electricity production.

The MFC operating temperature was held constant at 35 °C at a constant speed of 150 rpm. Anaerobic conditions within the anodic chamber were reached by minimizing the head space and bubbling nitrogen for at least 10 min prior to each culture.

The MFC performance was evaluated through the net amount of charge (*Q*) produced during each treatment, which was determined using the profile current vs. time (*I* vs. *t*) by the means of the following expression (Equation (1)):

(1) Q=∫I dt

and by the construction of the power curves (*P = V·I*) (employing the evaluation of polarization curve *V* vs. *I*) which allow for the determination of not only the maximum power (*P_máx_*) generated by the cell, but also the internal resistance which determines the relationship between the maximum power generated by the cell and the square of the intensity of the current (Equation (2)) [33].

(2) $$R_{int}=\frac{P_{máx}}{I^{2}}$$

### 3.2. Experimental Design and Statistical Analysis

To evaluate the effect of the surfactants on the MFC operation, Tween 80 as synthetic surfactant and surfactin as biological surfactant were separately evaluated in the anode chamber using *E. coli* DH5α. For each surfactant, three concentration levels were evaluated, namely (0.5, 5.0, and 10.0) times its CMC. The CMCs for the surfactin and tween 80 were 11.5 mg L^−1^, and 13.0 mg L^−1^, respectively [24,34].

Aiming to evaluate the impact of endogenous biosurfactant on current generation, a co-culture between *E. coli* as an electron producer and *L. plantarum* ATCC 814 as an in situ biosurfactant producer was tested with an MFC. Individual *E. coli* DH5α and *L. Plantarum* assays were conducted as controls to compare the effect of the co-culture.

### 3.3. Analytical Methods

Glucose was measured according to the glucose oxidase method [35]. Biomass was determined by measuring the optical density of a culture sample at 600 nm or by counting colony-forming units (CFU) using the microwell technique [36].

## 4. Conclusions

Microbial fuel cells are devices capable of achieving organic matter degradation while producing electricity. The presence of surfactants was found to be beneficial for current production; however, high concentrations of synthetic surfactants such as tween 80 could eventually adversely affect *E. coli* growth and so current production. The largest electric charge densities, 176.2 C m^−2^ and 166.1 C m^−2^, were achieved by using the tween 80 at 5.0 CMC and biological surfactant surfactin at 10.0 CMC, respectively. This was almost between 178.5 and 195.5% more than the current rendered by the control test without surfactant. The co-culture operation of the MFC using *E. coli* DH5α and *L. plantarum* allowed for achieving electrical charge density values 118.9% higher than the control with *E. coli* and 30% higher than the control with *L. plantarum*. It was evident that the presence of exogenous surfactants, as well as the production of endogenous biosurfactants through co-cultivation, led to significant increases in the maximum cell power output. The increase in power was attributed to a reduction in the internal cell resistance, likely due to an improved electron transfer rate resulting from enhanced cell permeability due to the surfactants. The empirical evidence collected here reveals two novel findings. First, the use of surfactin was found to enhance electricity production. Second, co-culturing *E. coli* and *L. plantarum* was found to enhance electricity production without the need for exogenous surfactants.

## Data Availability

Data are contained within the article.
